# Dr. Shearman on Chronic Debility

**Published:** 1824-09-01

**Authors:** 


*Observations Illustrative of the History and Treatment of Chronic Debility; the Prolific Source of Indigestion, Spasmodic Diseases, and various Nervous Affections.* By William Shearman, M.D. Member of the Royal College of Physicians, President of the Medical Society of London, Senior Physician to the Royal West London Infirmary, and Physician to the London Dispensary. 8vo. pp. 225, London, 1824.

The motto which Dr. Shearman has affixed to his work is very appropriate. "Et imbecillis, quo in numero magna pars urbanorum, omnesque pene cupidi literarum sunt, observatio major necessaria est, ut quod, vel corporis, vel loci, vel studii ratio detrahit, cura restituat." *Celsus.* Thus we see that congregated societies, over grown cities, sedentary habits, studious occupations, refinement of manners—in one word, high civilization, produced the same effects on the banks of the Tyber two thousand years ago, as we now see produced on the banks of the Thames. We every day observe a considerable proportion of our fellow creatures in a state which cannot be classed under the head of either health or disease. There is a less than ordinary vigour of the functions, and yet no cognizable or positive affection of any part. This state we are forced to designate *debility*, which, as Dr. S. justly observes, is sometimes as distressing or even as formidable as actual disease, and not unfrequently as difficult of removal. This state then, which may be considered as occupying a neutral ground between health and disease, has been chosen by our author for the subject of the volume under review.

Dr. S. sets out with a caution against confounding that debility or weak state of the constitution inherited from birth, with that deviation or decline from the usual and ordinary degree of strength, which is either the termination of disease, or produced by debilitating causes. These states of weakness are essentially different, and require very different methods of treatment. In considering the history and treatment of debility, it is necessary to keep in view the different kinds of debility which are presented to the practitioner. Weakness remaining after acute diseases is much more easily cured than weakness produced by the operation of debilitating causes, without the occurrence of actual disease. Our author notices an exception to this general rule which we consider a proof of his attentive and accurate observation. "In that particular species of catarrh, called influenza, there appears to be some exception to this rule; for, although the disease was, in every instance, of short continuance, yet, the debility produced by it continued, in some cases, for weeks, and even for months, and was with great difficulty removed."

We have, not unfrequently, remarked in our own practice, the great difficulty with which the constitutional powers of the patient are restored after attacks of this nature, whether epidemic, or not; and we cannot too strongly guard the inexperienced practitioner against any premature attempts being made to invigorate the patient by the administration of tonics, or stimuli. The same objection, it is true, will apply to every case of general debility, the effect of local disease, but with additional force and propriety, when the pulmonary system has been the seat of previous derangement. For the purpose of enabling us to appreciate justly the relative proportion of living power, expended by each particular system, or order of structure, in a state of health, and to estimate the deviations from such healthy state, as well as to comprehend the means of obviating the ill effects arising from such deviation, our author premises some general observations on the exertion and reproduction of this power, for which we must refer our readers to the work itself. Dr. S. studiously and judiciously abstains from any romantic speculations upon the nature of the living power; his object is "only to state obvious and ascertained facts, connected with the exhaustion and reproduction of this principle."

"Diseases of debility," says Dr S. "may be divided into those depending upon the increase of the mobility, and those depending upon the increase of the irritability of the system. In proportion to the weakness of the system, is the increase of these two properties of the body, irritability and mobility. By the increase of the former, parts are more readily excited to action, by stimuli applied to them; whether the natural stimuli, upon which their usual actions, in health, essentially depend, or external and adventitious stimuli; by the increase of the latter property, parts are more easily excited to action, by stimuli applied, not to themselves, but to other parts, in consequence of what has been called sympathy, or associated action. We are inclined to believe, that sufficient attention is not paid by many practitioners, in their treatment of disease, to the following facts, laid down by Dr. Shearman. A greater, or a more fatal error, cannot exist in the mind of the physician, than the belief that general and local debility proceed *pari passu.*"

"Even diseases of strong action may take place in persons of very weak habits; for it is not uncommon, when the system in general is weak, for some part of the body to act strongly. Weakness even gives an irregularity to the whole system, so as to give stronger action to one part in particular than to the others. It will sometimes happen, that when the habit is weak the arteries will act more powerfully; and this may even give occasion to active hæmorrhage, a disease of strong action, as from the lungs, intestines, or other parts of the body. Here, and in similar cases, although it is requisite not to neglect the appropriate treatment of the existing disease, it is essentially necessary to remove that state of debility which gave origin to, and maintains, the particular affection." 60.

It is justly observed by our author, that there are certain constitutional predispositions to particular diseases existing in some individuals, as to pulmonary consumption and scrofula: which diseases are more readily produced in a state of debility than they otherwise would be, and that frequently they are brought into action from this cause, in consequence of sufficient care not having been paid to support the vigour of the system. The progress of every disease is of course influenced by the existing debility, and the treatment requires a corresponding modification. In a very strong, and in a very weak habit, the same exciting cause may be productive of inflammation, but the disease will assume a very different character in the two constitutions. "In the former we shall usually have phlegmonous inflammation, with a strong tendency to suppuration; in the latter, erysipelatous inflammation, in which suppuration rarely, if ever, takes place when pure."

It does not come within the plan of Dr. Shearman's work to treat of the various modifications of particular diseases produced by chronic debility; but to offer some observations on the causes of that affection, and to endeavour to point out the most proper treatment of it, considered abstractedly and independently of any actual distinct disease with which it may at any time be combined. The direct and indirect causes of chronic debility are noticed, and the mode in which they act upon the human body pointed out. We agree in the following opinion of our author, and believe there are few practitioners who are not now aware that suppression of the menses is sometimes productive of consumption.

"It has been confidently asserted that pulmonary consumption is never the consequence of obstructed menstruation, but that in those cases where these two states are combined, the pulmonary disease has previously existed, and given rise to the obstruction. Every practitioner must have seen instances in which obstruction has taken place from some accidental cause, as cold, when no disease of the lungs had previously shown itself, and yet in some time after, the obstruction continuing, pulmonary disease has come on; whereas, in cases precisely similar, but wherein the menses have speedily recurred, no such disease has resulted." 94.*

The various debilitating causes which produce chronic debility by their primary and immediate operation are stated to be,

"1st, Various chronic diseases. 2dly, Long-continued evacuations. 3dly, Deficient supply of nourishment. 4thly, Inordinate use of stimulants or of sedatives. 5thly. Living in an impure atmosphere. 6thly, Warm climates." 101.

For the individual consideration of these several causes we must refer to the work.

The author now proceeds to speak of some of the most appropriate remedies, for the relief of these distressing ailments which are not more afflicting to the patient than perplexing to the practitioner. The same general principles obtain as in the treatment of other diseases. Chronic debility, the result of plethora, is, according to our author entitled to special attention, and for the removal of it many useful practical hints are suggested, particularly with regard to that common and obstinate state of weakness, which is the result of suppression of the menses in females.

"In the earlier stages of this affection, perhaps, the disposition to vascular contraction may be overcome by temporarily weakening the action of the sanguiferous system by an evacuation of blood, the utility of which will be in proportion, not to the quantity drawn, but to its immediate effects upon the vessels; it sometimes happening, even from a very small quantity suddenly taken, that such a change is produced in their action as to prevent a return of this morbid disposition, at least for some time, during which, means may be employed for removal of the original cause, the suppression." 150.

In strong and robust females, bleeding is particularly demanded, and

"In persons of more weakly frames, should we succeed, by blood-letting, in suspending for a time the disposition to inordinate contraction in the vascular system, an opportunity will be afforded of exhibiting stimulants and tonics to produce the natural discharge. When, however, this disposition has continued for a long time, is partly kept up by habit, and has produced a very great degree of debility, it becomes a question, whether blood-letting is at all admissible, and whether it will not aggravate, instead of lessening, the mischief?" 151.

* See Edinburgh Med. Journ. vol. vi. pp. 75,175. Med. and Phys. Journ. vol. xxiii. p. 519.

The combined exhibition of stimulants and tonics in these cases answers much better than medicines of either class, singly. Whatever medicines are selected will require to be variously combined and frequently changed.

Some interesting observations upon deranged digestion, and upon the principles upon which particular kinds of food should be selected, are made, and to which our limits prevent us from giving as much space as they merit. Sarsaparilla is considered by our author, one of the most valuable remedies which we possess, when we require a sedative to take off the morbid contraction of the blood vessels.

Various medicines are employed, with a view to diminish the irritability and mobility of the system, which are always morbidly increased in chronic debility. In our own opinion, the extr. conii, *if good*, is a remedy which will rarely fail to relieve the patient, and do credit to the physician. The use of opium, our author truly observes, in cases of chronic debility, requires considerable caution. Such is also the case with respect to wine.

Dr. S. does not enter into any discussion on the comparative merits of the various and well known tonic remedies; he offers only a few remarks on the general principles which should regulate their use. A succession of different tonics, and various combinations of them, are often required. A pure air is essentially necessary in chronic debility, and regular exercise, modified, of course, by the yet remaining powers of the patient. Anxiety of mind is the grand, the formidable, and the frequent opponent to the success of any remedial means. Every exertion must be made to remove it.

Dr. Shearman's work will be read with much benefit by the student, and junior practitioner. The subject upon which it treats is of such frequent occurrence, is productive of so much bodily and mental suffering, that we must consider ourselves indebted to our author, for the attention he has bestowed on its investigation. At the same time, we think Dr. Shearman has done much injustice to his own talents, in selecting a subject which no talent could render very productive. If the maxim of Hippocrates be true, that—"similia similibus curantur,"—it is, we think, equally true, that—similia similibus gignuntur. In other words Dr. Shearman has expended a good deal of his own strength in combating the debility of his subject.

